# Supplementary material for: Identification of a culturable fungal species and endosymbiotic bacteria in saliva of Aedes aegypti and Culex pipiens and their impact on arbovirus infection in vitro
Source: Parasit Vectors. 2025 Dec 17;19:40. doi: 10.1186/s13071-025-07158-3 (PMC12822050; doi:10.1186/s13071-025-07158-3)
Supplement: Supplementary file 1 — Supplementary material 1: Fig S1: Gel electrophoresis after PCR amplification. Fig S2: CFU of salivary bacteria by culturing on different agar plates. Fig S3: Bacterial loads of individual midgut and salivary glands of Culex pipiens and Aedes aegypti mosquitoes. Fig S4: Bacteria composition in mosquitoes variation according to life stage and sex. Fig S5: Survival rate of mosquitoes recorded for each treatment group. Table S1: Bacteria identified in the saliva of Aedes aegypti. Table S2: Bacteria identified in the saliva of Culex pipiens. Table S3: Sequencing input and output reads used for downstream analysis. [file 13071_2025_7158_MOESM1_ESM.docx]

# **Supplementary Information**

## **Supplementary Figures**

**Fig S1. Gel electrophoresis after PCR amplification.** DNA fragments were amplified with ITS3-4 (ITS) universal primers (350 bp) and a *Penicillium crustosum* specific primer pair (892 bp) on 3 fungal colonies tested.


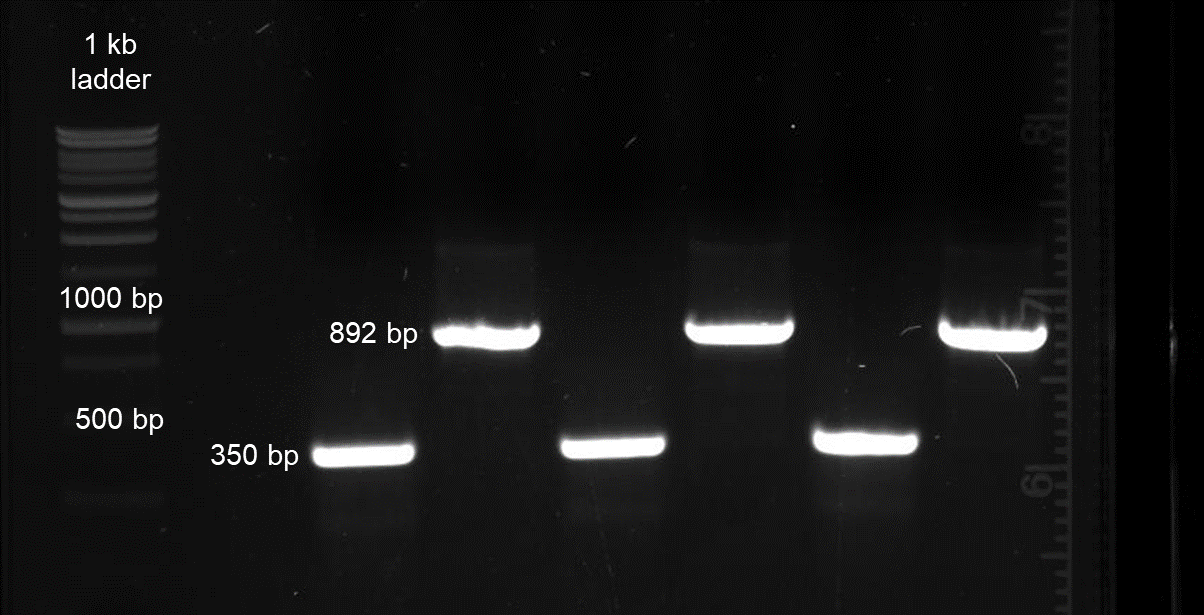


**Fig S2. CFU of salivary bacteria by culturing on different agar plates.** (A-B) Pooled saliva of *Aedes aegypti* cultured on LB, BHI and blood agar plates and incubated at 28°C under aerobic conditions. After 48 hours, colonies were counted, and CFU/mosquito was calculated for each agar plate for saliva from *Aedes aegypti*. Significant differences were demonstrated by the Kruskal-Wallis test (ns, p>0.05).


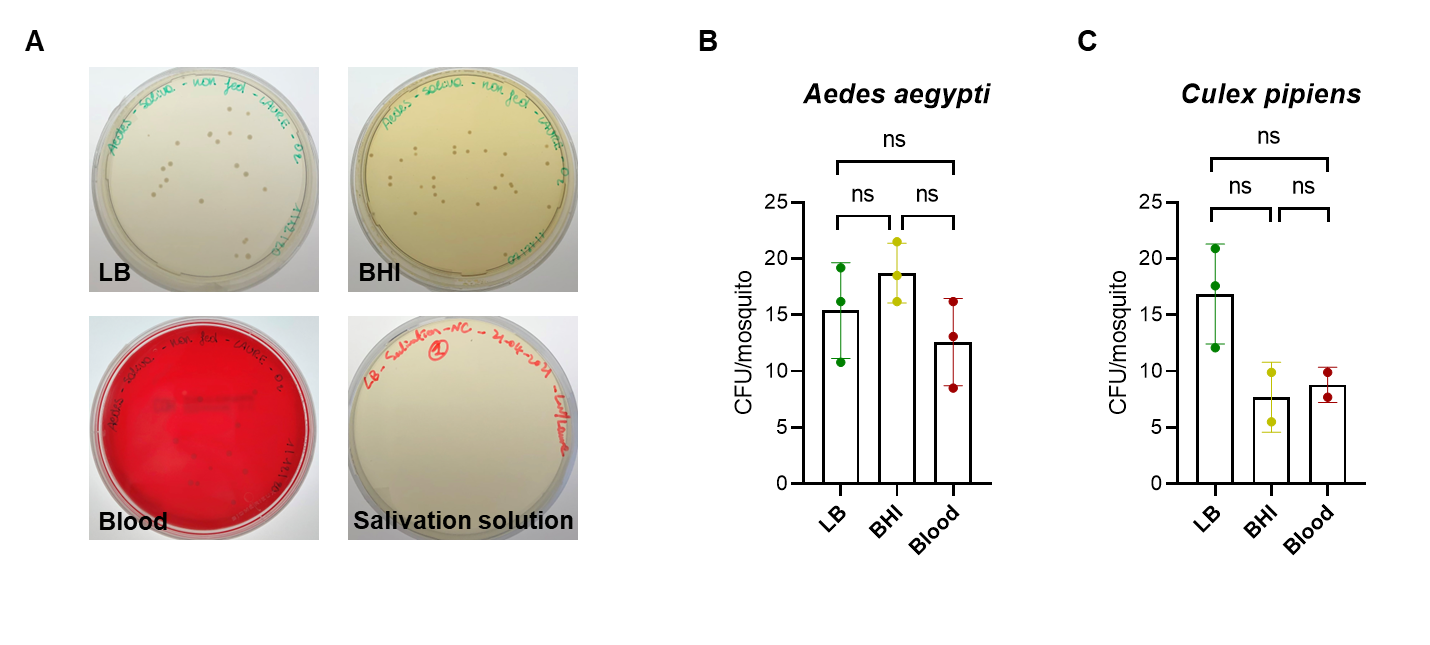


**Fig S3. Bacterial loads of individual midgut and salivary glands of *Culex pipiens* and *Aedes aegypti* mosquitoes.** The CFU read-out was performed by visual counting after culturing the homogenized tissues for 48h at 28°C on LB agar plates. Negative control (NC) consisted of the blank solution used as environment control for forced salivation. For the salivary gland samples, 50 µL of supernatant post-homogenization was used. For the midgut, the supernatant was diluted 10 to 100 times in sterile water post-homogenization before culturing 50 µL of the supernatant on the agar plates. SG = salivary glands. The lines represent the median values.


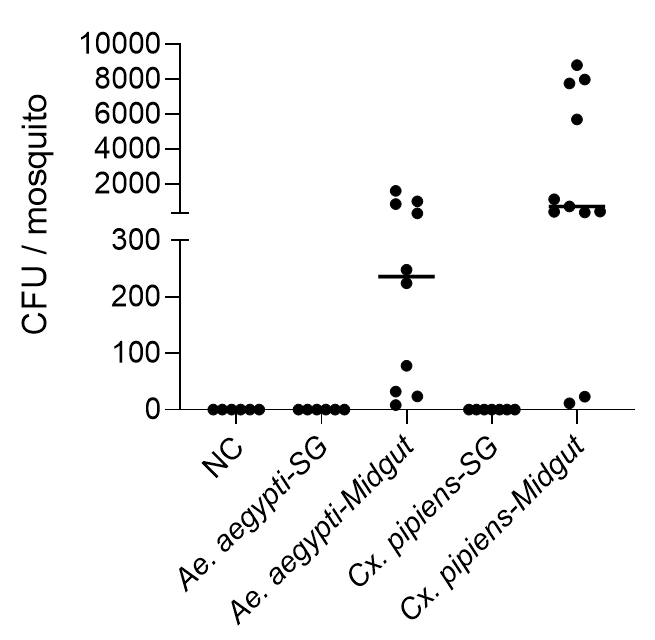


**Fig S4. Bacteria composition in mosquitoes variation according to life stage and sex.** Based on the 16S Sanger sequencing, number of colonies (%) per genus was calculated among pupae males; newly emerged males (D0); 2-week-old males; pupae females; newly emerged females (D0); and 2-week-old females.

**Fig S5. Survival rate of mosquitoes recorded for each treatment group.** Female *Aedes aegypti* adults (5-7 days old) were divided between three treatment groups: untreated control group (NT: only 10% sucrose), antifungal-treated (AF: 25 µg/mL of Fungin in 10% sucrose for 10 days) and antibiotic-treated (AB: serial dilution up to 200 units/mL of penicillin and 200 µg/mL streptomycin + gentamycin in 10% sucrose for 6 days).


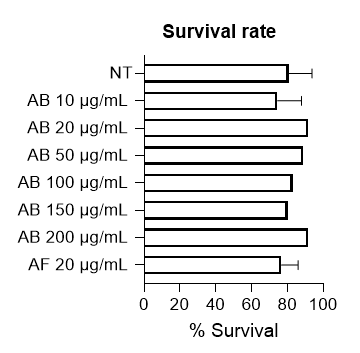


## **Supplementary Tables**

**Table S1. Bacteria identified in the saliva of *Aedes aegypti.***

| Agar type | Bacteria | % of sequenced colonies | Accession numbers |
| --- | --- | --- | --- |
| LB | *Klebsiella* spp. | 31 | MK691689; KX214770; CP087580; MH680856 |
|  | *Enterobacter* spp. | 16,9 | MK209686; OK326434; MN935016; MK764955 |
|  | *Asaia* spp. | 16,9 | MK391152; MK128664 |
|  | *Serratia marcescens* | 14,1 | KM091721; MH361385; CP055161 CP072199; MN788631; MT645673 |
|  | *Serratia nematodiphila* | 12,7 | MT409573; |
|  | *Microbacterium* spp. | 5,6 | MN922860; MT761105 |
|  | *Chryseobacterium* sp. | 2,8 | MT539177 |
| BHI | *Enterobacter* spp. | 60 | OK326434; MN935016 |
|  | *Klebsiella* spp. | 40 | MZ379584; KX214770 |
| Blood | *Enterobacter* spp. | 38,5 | MW435508; OK326434; MN935016 |
|  | *Klebsiella* spp. | 38,5 | MK691689; KX214770 |
|  | *Serratia marcescens* | 23 | CP055161; CP072199; MN788631 |

**Table S2. Bacteria identified in the saliva of *Culex pipiens.***

| Agar type | Bacteria | % of sequenced colonies | Accession numbers |
| --- | --- | --- | --- |
| LB | *Serratia marcescens* | 66,7 | KX904726; MH361385 |
|  | *Serratia nematodiphila* | 33,3 | MT409573 |
| BHI | *Serratia marcescens* | 100 | KX904726; MT645673 |
| Blood | *Serratia marcescens* | 100 | CP072199; MN788631; MT645673 |

**Table S3. Sequencing input and output reads used for downstream analysis.**

| Samples | input | filtered | denoised | merged | output |
| --- | --- | --- | --- | --- | --- |
| Midgut | 94352 | 76181 | 76096 | 75660 | 75660 |
| Midgut | 116483 | 94833 | 94736 | 94098 | 94098 |
| Midgut | 108028 | 88778 | 88722 | 88221 | 88221 |
| Midgut | 133391 | 109364 | 109311 | 108237 | 108237 |
| Midgut | 153228 | 115421 | 115286 | 114807 | 114807 |
| Midgut | 114668 | 91291 | 91188 | 90347 | 90347 |
| NC | 123743 | 97161 | 96946 | 96550 | 96550 |
| Saliva | 115967 | 92701 | 92360 | 91185 | 91185 |
| Saliva | 153093 | 124181 | 123137 | 121554 | 121554 |
| Saliva | 135960 | 105277 | 104738 | 104164 | 104164 |
| Salivary Glands | 134445 | 115265 | 115041 | 114074 | 114074 |
| Salivary Glands | 128798 | 106331 | 106062 | 105183 | 105183 |
| Salivary Glands | 147122 | 115252 | 114567 | 113634 | 113634 |
